# Seasonal Climate Effects Anemotaxis in Newly Emerged Adult *Anopheles gambiae* Giles in Mali, West Africa

**DOI:** 10.1371/journal.pone.0026910

**Published:** 2011-11-16

**Authors:** Nicholas C. Manoukis, Ibrahima Baber, Moussa Diallo, Nafomon Sogoba, José M. C. Ribeiro

**Affiliations:** 1 United States Pacific Basin Agricultural Research Center, Agricultural Research Service, United States Department of Agriculture, Hilo, Hawaii, United States of America; 2 Malaria Research and Training Center, Faculté de Médicine, de Pharmacie at d'Odonto-Stomatologie, Université du Mali, Bamako, Mali; 3 Section of Vector Biology, Laboratory of Malaria and Vector Research, National Institute of Allergy and Infectious Diseases, National Institutes of Health, Bethesda, Maryland, United States of America; University of California, Berkeley, United States of America

## Abstract

The direction and magnitude of movement by the malaria vector *Anopheles gambiae* Giles has been of great interest to medical entomologists for over 70 years. This direction of movement is likely to be affected by many factors, from environmental conditions and stage of life history of the mosquito to the existence of attractants in the vicinity. We report here the direction of movement of newly emerged *An. gambiae* in nature, around the village of Donéguébougou, Mali. We assessed the direction of movement for individual mosquitoes by placing them in a novel enclosure with exit traps oriented in the direction of the cardinal and intermediate points of the compass. We consistently found predominantly Southward directions of movement during 2009 and 2010, with an additional Eastward component during the dry season and a Westward one during the wet season. Our data indicate that wind has an important effect on the direction of movement, but that this effect varied by season: Average directions of movement were downwind during the dry season and upwind during the wet season. A switch in anemotactic response suggests that the direction of movement of *An. gambiae* relative to the wind immediately after emergence under varying conditions of humidity should be further investigated under controlled conditions.

## Introduction

*Anopheles gambiae* sl is the major vector of malaria in the African continent, where over 90% of the deaths from malaria occur [1]. For an individual female of this species to go from egg to actually transmitting the disease she must successfully engage in a long series of behaviors, starting with feeding and surviving in the aquatic habitat, through emerging and moving around the physical space surrounding the breeding site. Only after these steps will she seek a blood meal from a potentially infected human, digest this to produce a clutch of eggs and then find a breeding site to lay the eggs. If she has also acquired the malaria parasite this must develop in her mid gut and infect her salivary glands before she will be able to transmit malaria to another human during the next blood meal, usually a period of 12 days at 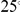
C.

Many links in the behavioral “chain” of *An. gambiae* from egg to blood feeding to transmitting the *Plasmodium* have been heavily researched, particularly those directly around host seeking. Excellent studies have been conduced on the role of carbon dioxide [2], lactic acid [3], 1-octen-3-ol [4], human sweat [5], dietary habits [6] and temperature and humidity [7] (among other factors) in host seeking, but these elements or other relevant parameters are almost never studied in relation to male mosquitoes (which do not blood feed but clearly play a role in maintaining vector populations) or to stages of the life history other than blood feeding. In this study we are interested in the choices facing an individual *An. gambiae* in the field immediately after emerging from the larval habitat. During those early moments as an adult, a mosquito is thought to seek shelter [8] to allow their exoskeleton to harden; blood feeding for females will not be a factor for several days, and so there are no infectious mosquitoes, but movement at this time will create the distribution of adult females that then may transmit malaria. Is the direction of movement of the new adults completely random? Do they attempt to approach a nearby village so they are near to mating sites [9] and sources of blood?

There is a known association between breeding sites and high mosquito density [10]; the link between mosquito density and parasite prevalence is often more complex [11], [12]. It is thought that emerging *Anopheles* probably move only a short distance from the breeding site based on observations in the laboratory of resting behavior after emergence [8] but the anthropophilic habits of *An. gambiae* are well known and may lead the newly emerged to move towards nearby human habitations for resting sites.

In order to test the direction of movement of recently emerged *An. gambiae* we constructed a large enclosure with exit traps in eight directions, oriented to the cardinal and intermediate points of the compass. The basic assumption of this experiment is that adults emerging at the center of the enclosure will be more likely to be caught in exit traps oriented in the direction they are trying to move, an idea supported by tests in the laboratory (see Materials and Methods).

Our results did not show a pattern of movement towards the village, but we did find consistent movement directions which are correlated with the direction of the wind and the season. During the wet season, under humid and warmer conditions, movement direction tended to be upwind. However, during the dry season, under dry and cooler conditions, movement directions were predominantly downwind. These results indicate that anemotaxis in *An. gambiae* should be closely examined under controlled conditions of varying humidity, temperature and wind speed.

## Results

We observed significant differences in the survival of adult *An. gambiae* post emergence during the dry and wet seasons. During the dry season almost all adults were dead by noon (about 18 h after the pupae were placed in the cages), and we never observed any living adults in the cages by 24 h after the start of any replicate. During the wet season, however, we caught adults in exit traps for up to 72 h after the start of the replicate.

During the wet season we checked exit traps every twelve hours (see Methods) but found that almost all mosquitoes found in exit traps were collected during the morning check rather than in the afternoon; and more were found in the second morning after the start of the replicate than the first. The mean number (SD) we collected in exit traps per enclosure on the first morning after the start of each replicate during the wet season was 43(30); the afternoon of the same day, 9(5); the following morning, 119(42). We only found a mean (SD) of 10(15) the third morning after the replicate started. Thus we have only considered the captures obtained in the morning after the start of the replicates for the dry season and for the two mornings after the start for the wet season in all subsequent analyses.

For the dry season, we found a mean(SD) of 71(29) adults per enclosure the morning following the start of the replicates. As mentioned previously, no living adults were ever found subsequent to that check during the dry season.

Average wind speed, measured from 6pm to 6am on nights when we ran the experiment, was more variable between years for the dry season than for the wet season. Relative humidity was much lower during the dry season than during the wet, as were average temperatures (Table 1).

**Table 1:** Weather on nights when experiments were conducted in Donéguébougou.

Date	Season	Wind Speed (m/s)	Gust (m/s)	Rel. Humidity (%)	Temperature (  C)
Apr 2009	Dry	0.36	1.62	31.20	31.27
Aug–Sep 2010	Wet	0.13	1.04	91.77	23.82
Mar–Apr 2010	Dry	0.09	1.20	30.98	30.32
Aug 2010	Wet	0.11	1.13	96.61	23.60

Averages from 18:00 to 06:00 on experimental nights. Gust = Average of nightly maximum wind speed.

We noted a very large difference in the number of adult mosquitoes caught in the corner traps of the cages compared to those along the sides of the enclosures. This difference was unexpected but very large: for the entire experiment we caught a total of 9267 adults in corner exit traps, compared with 350 in the other half of the exit traps which were along the sides of the enclosures. For all subsequent analyses we considered only the results from the corner cages.

We did not detect a significant difference in the number of males and females per exit trap (Wilcoxon signed rank test 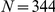, 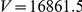, 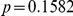). The data from our control cage show that just over 95% of the adults which emerged had done so by 22:30 on the first night of the experiment.

### Movement Direction in Field Trials

**Figure 1:**
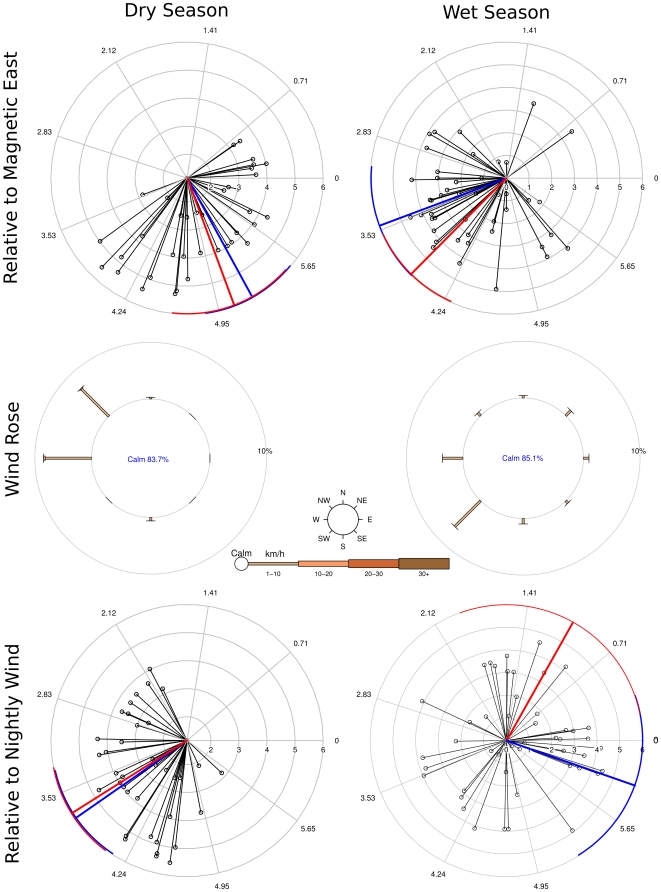
Individual dispersal vectors, seasonal and annual means and variances and wind roses. Plots in the left column are for the dry season and on the right for the wet season. The top row are dispersal vectors for each night and enclosure experiments were conducted (black lines with open circles) with the circular mean and variance of movement directions for 2009 in blue and 2010 in red. Vectors in this row are plotted relative to magnetic East at 0 radians (all angles measured counterclockwise). The middle row of plots are wind roses for nights when the experiment was conducted as measured from 18:00 to 06:00, again with East pointing to the right. The percent calm conditions is shown by the size of the center circle. Each branch represents the wind coming from that direction, with its thickness proportional to the speed of the wind (see scale at center). The length of each branch is proportional to the frequency of wind coming from that direction. The bottom row of the figure shows individual dispersal vectors plus means and variances relative the the mean wind direction on the night the experiment was conducted (upwind direction is 0 radians).

We calculated “dispersal vectors”, representing the direction and magnitude of the bias in movement directions (see Materials and Methods) for each enclosure and each night of the experiment, a total of 86 dispersal vectors. Of these, 17 and 24 were for the dry and wet seasons of 2009, respectively, and for 2010 the numbers were 21 and 24 for the dry and wet seasons. The direction and magnitude of each of all these dispersal vectors is given in Figure 1, which also shows the circular means and variances for each year and season.

In addition to the measured movement directions relative to the points of the compass given in the top row of Figure 1, we have also re-projected the the directions relative to the circular mean direction of the wind on the night the replicate was conducted (bottom row).

71 of the 86 dispersal vectors were of greater magnitude than 95% of the expected magnitudes from simulations of random directions of movement (see Materials and Methods). After removing those below that threshold, the number of dispersal vectors per season were 16 for dry 2009, 19 for wet 2009, 20 for dry 2010 and 16 for wet 2010. We used these 71 angles for all the inferential analysis that follows below.

We found that there was a significant departure from circular uniformity as estimated with the Hodges-Ajne “omnibus” test [13] within all the four seasons the experiment was conducted (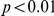 in all cases).

We found no statistically significant difference in the directions of movement between years for a given season (Watson-Williams tests: dry season 2009 vs dry season 2010 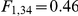, 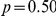; wet season 2009 vs. wet season 2010 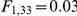, 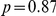). The same analysis showed no statistically significant difference in movement directions between years for a given side of the village (North or South): Marche vs Fulani (North) 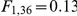, 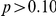; Cemetary vs. Grove (South) 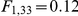, 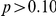.

Finally we conducted a two-factor Harrison-Kanji test (a circular analogue to a two-factor ANOVA [13]) to jointly estimate the effect of season and side of village on dispersal vectors. This test showed that both factors were statistically significant (Table 2). For side of village the predominant direction of movement for Northern and Southern sites was Southward: almost exactly South for the Northern sites and roughly South West for those in on the Northern side of the village. We observed almost no Northward movement at all.

**Table 2:** Effect of side and season on dispersal vectors.

	d.f.	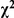	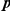
Side of village	2	8.00	0.02
Season	2	30.65	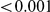
Interaction	1	3.48	0.96
Residual	66		

Harrison-Kanji two-factor test of the effect of side (North or South) and Season (Dry or Wet) on dispersal vectors.

## Discussion

Our results shed some light on the world of *An. gambiae* immediately after emergence from the larval habitat. The direction of movement of these new adult mosquitoes is far from random: we observed consistent Southward movement, with an Eastward component during the two dry seasons and a Westward one during the two wet seasons of research in Donéguébougou. The location of the village seemed to have no effect on the direction of movement, despite its relative proximity: though the two-factor test showed a significant effect of side of village on dispersal direction, we did not observe the difference to correspond to movement towards the village, i.e. the sites to the South of the village showed Southward rather than Northward movement. This indicates that the very first resting sites of *An. gambiae* are probably not tied to human habitations.

The leading explanatory factor in our study was the direction of the wind. During the dry season, the “Harmattan” winds are consistent and usually strong in Mali, coming from the North-West during night hours. The recently emerged adults showed a clear pattern of movement in the same direction as the wind during this season. During the wet season the pattern with respect to the wind was less clear, as the wind direction was more variable. However, the data suggest upwind movement. The difference between the seasons may indicate a switch from appetitive (active flight) to passive (drifting with the wind) [14] dispersal depending on environmental conditions, though there are additional explanations not excluded by our study but discussed below. We also note that any discussion of anemotaxis or movement in mosquitoes must be based on what is known about host-seeking behavior, which may not apply to the stage of the life cycle we are investigating.

Upwind movement is consistent with studies on mosquitoes and other insect species [15], [16] and with movement towards an attractant source [2], [17], [18], particularly for host-seeking mosquitoes [19]–[21]. Factors driving possible appetitive movement this early in the adult life cycle are unknown at present. However, if there is some attractant for the newly emerged mosquitoes during the dry season, it is probably not the village but rather something more ubiquitous; perhaps higher levels of carbon dioxide in the wind during the wet season could cause a seasonal difference, though we would expect lower CO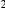 during the wet season due to plant growth.

Downwind movement is less commonly described in mosquitoes, though it has been suggested as an important component in host seeking: covering larger distance by moving downwind and moving upwind only when within close range [22]. Explanations for passive movement in host seeking mosquitoes are much less developed, but such behavior may be a common feature of non-feeding recently emerged adults under dry season environmental conditions, and perhaps for the same reason suggested by Gillies [22].

A simple difference in wind speed or gust strength between the seasons could explain the variation in direction of movement between seasons: newly emerged *An. gambiae* might be moving downwind during the dry season due to stronger average speeds or gusts compared to the wet season. However, our data indicate that wind speed was not consistently higher during the dry season, and the higher average gust strengths during those times don't seem sufficient to explain the highly consistent downwind movement of adults. For mosquitoes wind speeds below 1 m/s are thought to allow active movement [23], and though clearly this speed varies by species, appetitive movement should be possible during most times even in the dry season. In addition, strong winds are likely to cause the adults not take off at all [24], though variation from a higher to a lower wind speed may result in higher number of flights [25].

Another possibility is that a simple difference in age of emerged adults between seasons might explain the variation in anemotaxis. During the dry season all adults emerged and were caught in the same night- none survived to move the following evening. The wet season mosquitoes often survived to the next evening, and we indeed caught a larger number on the second morning compared with the first during those experiments.

We also note that the pupae used during the dry season were M-form from Niono, some 300 km away from our study site, while those used during the wet season were local and predominantly S-form (see Materials and Methods). Though the M-form makes up almost all the local population in Donéguébougou during the dry season, their numbers are very low. There may be a systematic difference in anemotactic response of early adults between the molecular forms, a possibility which could be further examined in the laboratory.

If there is a switch in anemotaxis not related to the molecular form but rather to the climate, the two major factors which could drive the change are temperature and relative humidity, both consistently lower during the dry season. Low relative humidity is known to lead to lowered activity levels in *An. gambiae* as measured by biting rate [26], [27]. These same studies indicate biting activity at temperatures around 20

C, so we do not think that temperature is limiting during the dry or wet seasons. Thus humidity is our leading explanatory factor for the variation in anemotaxis between seasons.

Few previous studies have considered anemotaxis when investigating movement by *An. gambiae* in nature, and there is only one release-recapture that we know of which also considered the age of the adults: the study of Gillies [28] in East Africa. His results did show both upwind and downwind movement during the NE and SE monsoon periods, but the wind directions were only the prevailing ones and humidity and temperature records are not reported. The same study also showed relatively lower distances of movement for 1 and 2 day old adults, around 0.4 km for the majority of recaptures.

We emphasize that our experiment concerned only the initial direction of movement, not the distance that any given adult *An. gambiae* may move following emergence. It may be that the distances covered are very small, and that the majority of new adults can be found in the morning directly around the breeding site in the hours after emergence [8]. While this strategy of very short-range movement might work during the wet season it would fail around any permanent water source during the dry season which is immediately adjacent and upwind from the shelter of a house or vegetation. It may be that short distance appetitive movement occurs during the wet season to nearby vegetation apart from humans, but passive movement during the dry season allows transport further and gives a better chance of survival.

Our results indicate that the direction of movement of *An. gambiae* relative to the wind immediately after emergence may vary depending on temperature and/or humidity. These should be further investigated under controlled conditions for newly emerged adults and also for older mosquitoes.

## Materials and Methods

### Study Site

**Figure 2:**
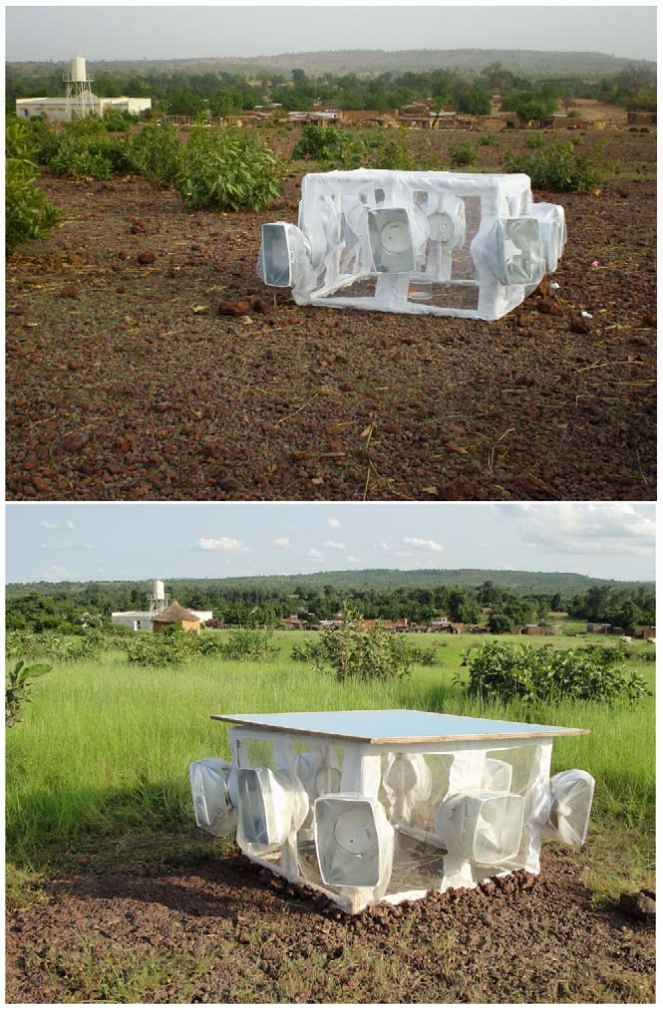
“Marche” site, North-West edge of Donéguébougou. Upper panel, dry season (April 2009); lower panel, wet season (August 2009). Note that the experimental enclosure pictured in the upper panel is not completely ready for the replicate: we always verified that the exit traps were at the same height before the start of the replicate.

We conducted our field experiments in the village of Donéguébougou, Mali. Donéguébougou is a village of 1345 inhabitants situated about 25 km north of Bamako, in the West Sudan Savana ecoregion, characterized by low grasslands and rolling hills. The area receives between 500 and 1000 mm of rainfall annually in a highly seasonal pattern, with almost all the precipitation occurring between May and October. There is an alternating dry season from November to April (see Figure 2). Our field experiment design included two dry season and two wet season experimental blocks, conducted in March–April (dry season) and August–September (wet season) of both years. We ran replicates for 6 and 8 nights during the dry and wet seasons of 2009 respectively, and 7 and 8 nights during the dry and wet seasons of 2010. We received permission from villagers and landowners to conduct experiments. Further details about the environment and human activities are given in [29].

Malaria transmission in this area is seasonal, coincident with rains and higher vector densities from June to November [30]. The major malaria vectors are in the *An. gambiae* species complex. The molecular and chromosomal form composition of *An. gambiae* in this area is known to shift with changes in climatic conditions between wet and dry seasons. The M molecular form (Mopti chromosomal form) is more prevalent, together with *Anopheles arabiensis* during the dry season, but they are gradually replaced by the S molecular form (first Savana then Bamako chromosomal forms) as precipitation increases [31], [32].

We selected four sites around the village to use for our experiment, which we named “Fulani” (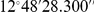
N 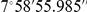
W) and “Marche” (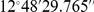
N 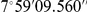
W) to the north and “Cemetary” (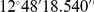
N 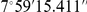
W) and “Grove” (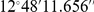
N 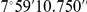
W) to the south. We selected these to be about 100 m from the edge of the village at the start of the experiment in March 2009.

### Emergence Enclosures

We developed a specialized enclosure for measuring the direction of movement of newly-emerged mosquitoes (pictured in Figure 2). These consisted of 57 cm cube enclosures made of PVC piping, with exit traps pointing in eight directions: in the center of each side and on each corner. Pupae were placed in these enclosures and moved into the exit traps as adults, usually overnight. Exit traps (opening diameter = 16 cm) were made from HVAC register boxes (GAF Materials Corp. Wayne NJ) with plastic funnels glued to the opening to prevent re-entry of mosquitoes from the trap back into the main enclosure chamber. The entire enclosure was covered with a custom-made screen which included zippers for closing the space around the exit traps. The exit traps were covered with screen fitted with elastic closures to prevent the escape of adults.

We designed these enclosures so that if mosquitoes were trying to go in a given direction they would be more likely to become caught in the exit trap pointing in that direction. Our observations of mosquitoes in the laboratory indicated that they would keep colliding with the netting or sides of the enclosure in a particular direction and move slightly along that surface, which makes it likely that they would be caught in the nearest exit trap. We validated this apparatus in an insectary using a carbon carbon dioxide close to one exit trap of the enclosure as an attractant and found significantly more adults in the exit trap near the source than in the others. We constructed and utilized a total of three such enclosures for the experiment presented here.

### Field Experimental Procedure

In order to obtain sufficient numbers of pupae for conducting multiple enclosure trials per night we collected blood-fed, semi-gravid and gravid *An. gambiae* from our field site in Donéguébougou by mouth aspiration during the wet season and allowed them to lay eggs in the MRTC insectary in Bamako. We raised the larvae until the pupal stage in the insectary. During the dry season the number of adult mosquitoes in the village was very low, so we collected from the irrigated region of Niono then followed the same rearing procedure in the laboratory. We note that during the wet season in Donéguébougou the *An. gambiae* population consists of mostly S molecular form individuals (Savana and Bamako chromosomal forms) while during the dry season the M molecular form predominates (Mopti chromosomal form) [31], [32]. *An. gambiae* from the irrigated area of Niono are very nearly 100% of the M molecular form [33].

On the morning of a replicate we would count pupae starting at around 08:00 h and sort them into batches of 100 individuals. Around 15:00 h we would mix the pupae (from different rearing trays) and segregate them into larger containers (with about 400 mL of water), one per enclosure and a fourth as a control group. The control group was around 100 pupae (minimum = 75, max = 120, mean = 96, SD = 10) in about 150 mL of water held in a growth chamber (BioQuip Products, Rancho Dominguez CA) next to our portable weather station at the field site. We checked this control growth chamber every 30 min–1 hour until at least 23:00 h to check for the time of emergence of our pupae.

Weather conditions were recorded using a Kestrel 4500 portable weather station with weather vane attachment (Nielsen-Kellerman, Boothwyn PA) mounted on a tripod at our field station (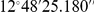
N 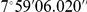
W). The device was set to record climatic conditions from 18:00 h to 06:00 h on the nights when we conducted the experiment. We did not record climatic conditions within the enclosures directly.

By about 17:30 h we had set up experimental enclosures in three of the four available sites, locations randomized between nights. The number of pupae varied between experimental nights, from a minimum of 200 to a maximum of 470 per cage (mean = 345, SD = 83). All enclosures were oriented with either a corner or a side trap pointing North on a per night basis (order randomized). The locations to which particular enclosures were deployed were also randomized.

The morning following the start of the replicate we checked each of the exit traps in each of the enclosures starting around 06:00 h. We used a mouth aspirator to remove any adult mosquitoes in each exit trap. When possible we counted the number of males and females in each exit trap in the field, though if numbers were high or weather conditions were unfavorable we would put the adults into collecting cups and take them back to the laboratory in Bamako for counting.

### Measuring Movement Direction Based on Enclosure Data

The data resulting from the enclosure experiment were counts of male and female adult *An. gambiae* found in each corner exit trap the morning or mornings after the start of each replicate. In order to analyze these we had to calculate the direction and magnitude of any differences between traps in the number of adults, which we were able to summarize as a single pair of numbers for each replicate (night/enclosure) by calculating a “dispersal vector” which had the direction (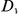) and magnitude (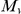) of the differences in counts of adults in each exit trap. We did this by considering each mosquito caught in a given trap to add a unit length to a vector pointing in that direction, and then used component addition to calculate the dispersal vector for the whole enclosure/night. When the corner cages were oriented in the cardinal directions we calculated the 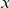 and 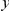 components as follows:
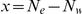


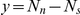
Where 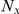 is the number of adults caught in the trap pointing in the magnetic direction 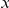. When the enclosures were oriented in the intermediate directions, we calculated the components as follows:
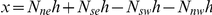


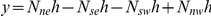
Where 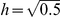. Finally, we calculated the dispersal vectors as:
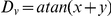


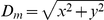



### Statistical Analysis

We produced basic descriptive statistics for the field cage data using the CircStats package in R [34], and further inferential tests using CircStat under Matlab [13]. One problem presented by our data when using inferential statistics is that methods for handling circular data analyze angles but not magnitudes of vectors on the circle. We had to determine which magnitudes were significant for inclusion in any analysis of angles and their relationship to explanatory variables such as the wind or the location of the village. In order to determine significant vector magnitudes for use in the inferential analyses only, we generated 1000 simulations of 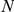 mosquitoes moving into random corner exit traps (random directions) for each enclosure/night of the real experiment, where 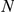 is the total number of mosquitoes that were caught in exit traps in that enclosure/night. We then used the dispersion vector magnitude from the simulation to determine if the observed vector magnitude each night exceeded the value of 95% of the random simulations. Note that this measure of “significant” vector magnitudes would exclude results with no significant directionality and likely also those with more than one major direction of movement.
